# 

**Published:** 1891-03

**Authors:** 


CONTENTS.

Vol. 38. No. 3.

| | PAGE. |
|---|---|
| Our Prison Systems | 49 |
| The Hygiene of Motherhood | 51 |
| A "Cold in the Head" | 56 |
| Impure Air | 56 |
| Edith Thomas's Blindness | 57 |
| Children | 58 |
| Second Sight | 58 |
| Nutritive Value of Foods | 60 |
| Effect of Climatic Changes | 60 |
| Remarkable Psychic Transformation of Mary Vennum | 61 |
| A Finnish Bath | 62 |
| Hygiene of the Eye | 63 |
| Pneumonia | 64 |
| Light Weight Bedding | 66 |
| Ancient Breathing Exercises | 66 |
| Miscellaneous | 67 |
| Talleyrand | 67 |
| Alcoholic Prescriptions | 68 |
| Literary | 71 |
| Now and Then I Question Me | 72 |

INDEX TO ADVERTISEMENTS OF HALF A PAGE AND OVER.

| | PAGE. |
|---|---|
| Horsford's Acid Phosphate | I |
| Lydia E. Pinkham Med. Co. | II |
| Geo. P. Rowell & Co | III |
| Hall's Journal Premiums | IV |
| Stonington Line | V |
| Ruckel & Hendel | VII |
| Ayer's Sarsaparilla | VII |
| House and Stable Pub. Co | IX |
| Aerated Oxygen Comp'nd Co. | X |
| Herba Vita | XI |
| Ladies' Home Companion | XII |
| Turf, Field & Farm Ass'n | XIII |
| Royal Baking Powder | XV |
| I. S. Johnson & Co | XV |

